# Socioemotional self- and co-regulation in functional seizures: comparing high and low posttraumatic stress

**DOI:** 10.3389/fpsyt.2023.1135590

**Published:** 2023-05-15

**Authors:** Nicole A. Roberts, Lucia Dayana Villarreal, Mary H. Burleson

**Affiliations:** School of Social and Behavioral Sciences, Arizona State University, Phoenix, AZ, United States

**Keywords:** functional seizures, psychogenic nonepileptic seizures, emotion regulation, posttraumatic stress disorder, affectionate touch, social connection

## Abstract

Functional seizures (FS) are seizure-like symptoms without electroencephalogram (EEG)-based epileptic activity. Those with FS often show emotion-related dysfunction and disrupted interpersonal relationships, in which posttraumatic stress disorder symptoms (PTS) may play a role. We sought to better understand trauma comorbidities and socioemotional processes in FS, including affectionate touch, a form of social connection linked to emotion regulation and awareness. We administered questionnaires online to a community sample of 89 trauma-exposed FS participants (FS diagnoses were self-reported), 51 with and 38 without clinical-level PTS (FS-PTShi, FS-PTSlo) and 216 seizure-free matched trauma-exposed controls (TCs), 91 with and 125 without clinical-level PTS (TC-PTShi, TC-PTSlo) per the Posttraumatic Stress Disorder Symptom Checklist (PCL). As hypothesized, both FS-PTShi and FS-PTSlo reported more emotional avoidance (Brief Experiential Avoidance Questionnaire), more emotion regulation difficulties (Difficulties in Emotion Regulation Scale), and more perceived stress (Perceived Stress Scale) than PTS-matched counterparts. FS-PTShi also reported less reappraisal (Emotion Regulation Questionnaire), more loneliness (UCLA Loneliness Scale), and less frequent affectionate touch (Physical Affection Scale) during waking and surrounding sleep than TC-PTShi, whereas FS-PTSlo and TC-PTSlo did not differ. Neither FS group differed from PTS-matched controls in emotion suppression (Emotion Regulation Questionnaire) or comfort with social touch (Social Touch Questionnaire). Among FS, FS-PTShi reported more difficulties than FS-PTSlo on nearly all measures (non-significant trend for social support). Findings underscore potential synergistic effects of FS and PTS clinical symptoms in shaping experiences of one’s emotions and social world, suggesting fostering meaningful connections with others, including via affectionate touch, is an important treatment target.

## 1. Introduction

Functional seizures (FS) resemble epileptic seizures behaviorally, but occur without concurrent electroencephalographic (EEG) epileptiform activity (1). This condition, also called psychogenic non-epileptic seizures (PNES), falls under the larger category of functional neurological disorders [FND; (2)], while specifically categorized as a “dissociative neurological symptom disorder” in *ICD-11* (3) or “conversion disorder (functional neurological symptom disorder)” in *DSM-5* [(4); see also (5, 6)]. As diagnostic and clinical entities, FND are now understood not only in terms of absence of expected neurological indicators, but also as conditions with known and emerging biopsychosocial correlates; for example, emotion processing disruptions are important vulnerabilities, and bodily states may be interpreted or categorized/labeled as somatic rather than affective [models in FND, (7–11); in FS, (12)]. FS is associated with high prevalence of trauma, especially violence exposure and experiences (5, 13, 14); this is a predisposing vulnerability to FS and its social and emotional correlates (see below). Alongside emotion processing-related challenges (15, 16), those with FS may experience conflictual interpersonal relationships (17, 18) and limitations in social functioning due to not working or driving, or concerns about having an FS episode (19). Interpersonal difficulties impact patients’ personal and family life (20), with implications for patient-provider communication (21). Nevertheless, FS individuals describe strong social engagement and desire to be with others (22), reporting larger social networks than individuals with other motor FNDs (23). We investigated experiences in FS of emotion regulation and social connection, including affectionate touch, while aiming to disentangle possible influences of traumatic stress symptoms and mental health comorbidities.

Emotion regulation can be viewed as a multi-faceted, dynamic process, beginning with deliberate or implicit decisions to engage versus avoid emotional situations, to reappraising situations to lessen their emotional impact, through outwardly expressing versus suppressing emotions [i.e., *process model* of emotion regulation; (24, 25)]. Emotion regulation difficulties can be operationalized as perceived (in)ability to handle feelings of upset (26); difficulties attending to or being aware of feelings is a related but separate facet (27). A review (28) examining studies of FS and emotion through a process model lens found that trouble identifying and describing emotions, and tendency to avoid emotions, were consistent features of FS—perhaps more so than for other FND [e.g., greater alexithymia in FS than functional movement disorder; (29)]—whereas extent of emotion regulation difficulties was less consistent, reflecting heterogeneity among emotion-related domains and within FS [e.g., high and low emotion dysregulation subgroups; (30, 31)].

Emotion regulation in FS in social contexts has received relatively less attention. Because emotions and emotion regulation often unfold within relationships, “co-regulation,” or affect (dys)regulation via other people, is important (32). Furthermore, in both clinical and community samples, feelings of belonging and social connection (or its lack, namely loneliness), can profoundly impact morbidity and mortality (33, 34). In FS, quality relationships (e.g., friendships) predict better prognosis (35) yet may be compromised by FS-related interpersonal distress [e.g., feeling invalidated or dismissed (36)] or struggles processing socio-emotional information (37). The physical component of social connection—affectionate touch—can itself regulate affect and bridge self-and other regulation (38). To our knowledge, this remains unexplored in FS.

As noted above, previous studies of FS have found alterations in *socioemotional regulation* processes. These FS-linked alterations include greater difficulties with emotional awareness and regulation (39), heightened preconscious attention to social threat (37), greater anxious arousal and other clinical symptoms (40) and related physiological correlates [e.g., lower high-frequency heart rate variability (41, 42), higher cortisol (37)]; yet, these in large part are accounted for in the studies cited by interpersonal trauma and posttraumatic stress reactions. In FS and other FND, adverse life events such as early childhood maltreatment are associated with difficulty forming trusting social relationships (43), and trauma and posttraumatic stress are related to lower social network size [and associated neural correlates; (23)]. Understanding socioemotional processes in FS necessitates considering links with frequently-comorbid conditions (e.g., posttraumatic stress disorder [PTSD]) that also are characterized by socioemotional dysfunction (44–46). In previous research, for example, subgroups of FS individuals with versus without PTSD symptoms showed worse socioemotional and symptom profiles (47–49). In other studies, emotion regulation difficulties were not uniformly greater in FS than in a trauma control group with clinical levels of PTSD symptoms (41, 42). This underscores the need to consider areas of overlap and distinction among potential FS subgroups, in addition to comparing with appropriate control groups.

In the present study, therefore, we investigated whether self-reports of emotion-related processes and interpersonal experiences suggested more problems among FS than controls after accounting for prior trauma exposure and posttraumatic stress reactions; we did this by matching FS groups based on PTS symptoms and overall mental health. We included measures that captured different kinds of affect regulation strategies (24–26, 50) and that reflected self-and co-regulation of affect. We assessed not only emotional avoidance, awareness, and regulation difficulties and tendencies, but also sense of connection to others psychologically and physical connection in social and partner relationships. We were especially interested in affectionate touch (including touch surrounding sleep, or “sleep-touch”), which is linked to affect regulation and relationship closeness (51–53), social and health benefits (38, 54), and emotional and somatic/body awareness (55), which are disrupted in FS (56).

We compared those with FS to seizure-free trauma-exposed controls (*TCs*) matched in posttraumatic stress symptom (PTS) levels, either at or above (*PTShi*) versus below (*PTSlo*) a clinical cut-off. We hypothesized that (H1) FS with high PTS versus controls with high PTS, (H2) FS with low PTS versus controls with low PTS, and (H3) FS with high PTS versus FS with low PTS would report more difficulties on indicators of socioemotional regulation.

## 2. Methods

### 2.1. Participants

Participants were 305 individuals with prior trauma: 89 with FS and 216 TCs. Inclusion requirements were as follows: age 18 or more years; for FS, self-reported diagnosis of FS (see below); for TC, self-reported exposure to traumatic event(s) and no prior seizures/seizure-like symptoms. We separated participants into “PTShi” and “PTSlo” subgroups using PTSD symptom scores (see below), yielding four groups: FS-PTShi (*n* = 51), FS-PTSlo (*n* = 38), TC-PTShi (*n* = 91), and TC-PTSlo (*n* = 125). Sample characteristics are in Table 1. Trauma characteristics are in Supplementary Tables 1, 2.

**Table 1:** Demographic and clinical characteristics by group.

Variable	FS-PTShi		FS-PTSlo		TC-PTShi		TC-PTSlo		*Χ*^2^ (df)	*p*-value
	*n*	%^a^	*n*	%^a^	*n*	%^a^	*n*	%^a^		
**Demographics**										
Gender/sex									5.6 (6)	0.513
Male	8_a_	15.7	4_a_	10.5	9_a_	9.9	16_a_	12.8		
Female	40_a_	78.4	34_a_	89.5	80_a_	87.9	107_a_	85.6		
Transgender	3_a_	5.9	0_a_	0	2_a_	2.2	2_a_	1.6		
**Ethnicity/race**									36.6 (15)	<0.001
Asian	1_a_	2.0	0_a_	0.0	3_a_	3.3	3_a_	2.4		
Black/African/Caribbean	1_a_	2.0	0_a_	0.0	9_a_	9.9	13_a_	10.4		
Hispanic/Latinx	6_a_	11.8	1_a_	2.6	13_a_	14.3	21_a_	16.8		
Native American or Alaska Native	0_a_	0.0	2_a_	5.3	3_a_	3.3	1_a_	0.8		
White	43_a_	82.4	35_a_	92.1	52_b_	57.1	70_b_	56.0		
Mixed	1_a_	2.0	0_a_	0.0	11_a_	12.1	17_a_	13.6		
**Ethnicity/race (Non-white vs. white)**									26.5 (3)	<0.001
Non-white	9_a_	17.6	3_a_	7.9	39_b_	42.9	55_b_	44.0		
White	42_a_	82.4	35_a_	92.1	52_b_	57.1	70_b_	56.0		
**Subjective income category**									35.6 (12)	<0.001
Lower	20_a_	42.6	14_a_,_c_	36.8	15_b,c_	16.5	15_b_	12.1		
Lower middle	10_a_	21.3	9_a_	23.7	32_a_	35.2	31_a_	25.0		
Middle	14_a_	29.8	11_a_	28.9	38_a_	41.8	59_a_	47.6		
Upper middle	3_a_	6.4	4_a_	10.5	4_a_	4.4	14_a_	11.3		
Upper	0_a_	0	0_a_	0	2_a_	2.2	5_a_	4.0		
**Relationship status**									4.2 (3)	0.244
Not in relationship	17_a_	33.3	11_a_	28.9	26_a_	28.6	26_a_	20.8		
In relationship	34_a_	66.7	27_a_	71.1	65_a_	71.4	99_a_	79.2		
	*M*	SD	*M*	SD	*M*	SD	*M*	SD	*F* (df)	*p*
Age	37.2_ab_	12.6	40.8_a_	12.7	32.0_c_	6.9	35.9_b_	8.5	8.42 (3,293)	<0.001
Years of education	14.9_a_	2.8	15.3_a_	3.0	15.0_a_	1.7	15.5_a_	3.9	0.64 (3,286)	0.591
**Clinical characteristics**										
Posttraumatic stress symptoms (PCL-5)	54.3_a_	12.5	16.2_b_	9.8	52.4_a_	9.5	14.9_b_	9.4	362.43 (3,302)	<0.001
Number of traumatic events reported	3.5_a_	1.9	2.2_b_	1.6	3.3_a_	1.7	2.3_b_	1.3	12.41 (3,302)	<0.001
Mental health inventory (MHI-5)	4.2_a_	0.9	3.0_b_	1.0	3.8_a_	1.1	2.6_b_	0.9	39.29 (3,286)	<0.001
Dissociative experiences scale (DES-II)	29.7_a_	18.3	15.2_b_	12.8	22.8_c_	14.4	13.1_b_	10.1	20.67 (3,286)	<0.001
Age of seizure onset	27.7	14.6	34.2	14.1	–	–	–	–	4.38 (1,86)	0.039
Duration of seizures (years)	9.5	10.7	7.0	8.5	–	–	–	–	1.35 (1,86)	0.248
Seizure frequency^b^	2.5	0.6	2.5	0.6	–	–	–	–	0.12 (1,88)	0.726
Seizure severity^b^	2.5	0.9	2.8	0.7	–	–	–	–	4.77 (1,88)	0.032
Seizure impact (IES)	3.4	0.6	3.1	0.9	–	–	–	–	3.90 (1,71)	0.053
	*n*	%^a^	*n*	%^a^					*Χ*^2^ (df)	*p*-value
**Most recent seizure occurred within**									7.5 (6)	0.277
Last 24 h	18_a_	35.3	14_a_	36.8						
Last week	20_a_	39.2	9_a_	23.7						
Last 2 weeks	5_a_	9.8	2_a_	5.3						
Last month	3_a_	5.9	6_a_	15.8						
Last 6 months	2_a_	3.9	5_a_	13.2						
Last year	1_a_	2	0_a_	0						
More than a year ago	2_a_	3.9	2_a_	5.3						

Values or means that do not share a subscript are significantly different at *p* < 0.05 or less. IES, Impact of Epilepsy Scale. Seizure characteristics compared only between FS-PTShi and FS-PTSlo.

a % = percentage within participant group (column).

b Single item (frequency: 1–3 scale; severity: 1–4 scale).

FS participants were recruited via partnering neurologists/neuropsychologists and websites/social media [e.g., FNDHope.org, NEAD.org (nonepileptic attack disorder), Arizona Epilepsy Foundation, Northeast Regional Epilepsy Group]. FS sample inclusion was based on participants’ self-reported diagnosis of FS or probable FS and questions to corroborate their diagnosis/symptoms (e.g., video-EEG results or physician reports; see Supplementary material). Trauma-exposed controls were recruited from a large university research participation pool “if they had a prior major stressful or traumatic event(s).”

We also created a subsample of FS participants (30 FS-PTShi, 23 FS-PTSlo) including only those who reported both (1) EEG/video-EEG results indicating FS [see (57, 58)], and (2) an FS diagnosis with which they agreed. We report how results with this more restrictive FS subsample compared with the full sample.

### 2.2. Measures

#### 2.2.1. Demographics

Age, gender, racial/ethnic background, education level, socioeconomic status, and marital/relationship status were recorded.

#### 2.2.2. Seizure frequency and severity

Seizure frequency was assessed with a single item coded into 3 categories: 1 = seizure-free for 1 year or more, 2 = monthly or less than monthly, and 3 = daily or weekly. Seizure severity was assessed with a single item: “Overall, how severe have your seizures or seizure-like episodes been in the past year?” Options were 1 = *very mild*, 2 = *mild*, 3 = *severe*, and 4 = *very severe*.

#### 2.2.3. Seizure impact: Impact of Epilepsy Scale (IES)

Participants rated 8 items (0 = *not at all* to 4 = *a lot*) assessing seizure symptom impact in multiple domains (e.g., work, social relationships) (59). Reliability was excellent: Cronbach’s *α* = 0.91.

#### 2.2.4. Trauma type(s): adverse Life Events Checklist (LEC)

We used an abbreviated Life Events Checklist (LEC; 60). Participants selected from 8 traumatic events which they experienced, and could write in experiences not listed. The LEC comports with PTSD-relevant criteria per diagnostic clinical interviews (60).

#### 2.2.5. Posttraumatic symptoms: PTSD symptom checklist for *DSM-5* (PCL-5)

Participants rated 20 items (0 = *not at all* to 4 = *extremely*) (PCL-5; 61). Thirty-seven early participants completed the 17-item PCL Specific Event version for *DSM-IV* [PCL-S; (62)], where items are rated from 1 = *not at all* to 5 = *extremely* with respect to the event that “stuck with them the most.” We used a crosswalk procedure (63) to convert summed PCL-S scores to summed PCL-5 scores. We used a clinical cut-off of 33, at or above which indicates a probable PTSD diagnosis (64), to create PTShi and PTSlo groups. [*α*: PCL-5 = 0.95, PCL-S (before crosswalk conversion) = 0.97].

#### 2.2.6. General mental health symptoms: Mental Health Inventory-5 (MHI-5)

Participants rated 5 items from 1 = *none of the time* to 6 = *all of the time* to indicate how much of the time during the last month they felt a certain way (e.g., “been a very nervous person”; “been a happy person,” reverse-scored) (65). Higher MHI-5 scores reliably predict clinical diagnoses, especially depression and anxiety (65). (*α* = 0.86.)

#### 2.2.7. Dissociative symptoms: Dissociative Experiences Scale-II (DES-II)

Participants rated 28 items on a 0% (*never*) to 100% (*always*) scale in increments of 10%, which were recoded from 1 (0%) to 11 (100%) for analysis (66). (*α* = 0.93.)

#### 2.2.8. Difficulties in emotional awareness and emotion regulation: Difficulties in Emotion Regulation Scale-Short Form (DERS-18)

Eighteen items reflecting six emotion regulation difficulty dimensions were rated using a 5-point Likert scale (1 = *almost never* to 5 = *almost always*) (26, 67). Total scores were computed by averaging 15 items (Cronbach’s *α* = 0.93). The three remaining items from the emotional awareness subscale were examined separately (*α* = 0.85).

#### 2.2.9. Emotional avoidance: Brief Experiential Avoidance Questionnaire (BEAQ)

Fifteen items were rated from 1 = *strongly disagree* to 6 = *strongly agree* (68). (*α* = 0.86.)

#### 2.2.10. Situational reappraisal and expressive suppression: Emotion Regulation Questionnaire (ERQ)

Six reappraisal subscale items (think about situation differently) and 4 expressive suppression subscale items (hide outward emotional display) were rated from 1 = *strongly disagree* to 7 = *strongly agree* (69). (*α*: reappraisal = 0.91; suppression = 0.80.)

#### 2.2.11. Perceived stress: Perceived Stress Scale (PSS)-Short Form

Four items measured perceived stress versus ability to manage it (0 = *never* to 4 = *very often*) (70). (*α* = 0.77.)

#### 2.2.12. Perceived social support: Interpersonal Support Evaluation List-Short Form (ISEL)

Two items each from the appraisal, tangible, and belonging support subscales were rated from 0 = *definitely false* to 3 = *definitely true* (71, 72). (*α* = 0.79.)

#### 2.2.13. Loneliness: UCLA Loneliness Scale-Revised-Short Form (UCLA-R)

Four items are rated from 0 = *never* to 3 = *often;* higher scores reflect greater loneliness (73). (*α* = 0.67.)

#### 2.2.14. Comfort with social touch: Social Touch Questionnaire (STQ)

We used an 18-item STQ version to measure touch-related attitudes and behaviors (e.g., “I’d be happy to give a neck/shoulder massage to a friend if they are feeling stressed”). Items were rated from 0 = *not at all* to 4 = *extremely*; higher scores indicated greater comfort with touch (74, 75). (*α* =0.88.)

#### 2.2.15. Affectionate touch frequency: Physical Affection Scale (PAS)

Eight items were rated for frequency (0 = *never* to 4 = *almost daily*) of affectionate touch with current partner or, if not presently in a relationship, with most recent partner (38, 76). (*α* = 0.91.)

#### 2.2.16. Affectionate touch surrounding sleep: “Sleep-touch”

Participants rated a single item, “How much do you and your spouse/partner ordinarily touch each other while sleeping in the same bed?” (0 = *not at all* to 4 = *very much*) (53).

### 2.3. Procedure

The study was approved by the university’s institutional review board. All procedures followed APA ethical standards for protection of human subjects. Participants completed measures reported here as part of a larger survey administered via a secure website, www.Surveymonkey.com. A link to the consent form and survey was provided on the recruitment materials. At the end of the survey, FS participants could opt to enter contact information (via a separate link) to receive a $35 giftcard (early participants) or be entered into a $35 giftcard drawing (later participants). Control participants received research participation credit.

### 2.4. Data analysis

We tested our hypotheses comparing FS individuals to trauma-matched controls (FS-PTShi to TC-PTShi, FS-PTSlo to TC-PTSlo) and comparing subgroups within FS (FS-PTShi to FS-PTSlo) with planned comparisons (*F*-tests) using Bonferroni correction to adjust for multiple comparisons, and with age as a covariate. We report effect size using partial eta squared (*η_p_*^2^). We conducted parallel analyses with an FS subsample constructed with more restrictive inclusion criteria (see Participants).

## 3. Results

### 3.1. Descriptive results: demographic and clinical characteristics

The following individuals were excluded before reaching the final 305-participant sample: 12 FS with inconclusive seizure/seizure-like event information; 31 TCs reporting prior seizures; 22 FS reporting no prior trauma; 28 FS and 33 TCs not completing the PCL; 2 FS participants over age 65 (to better age-match groups) and 8 TCs not reporting age; 12 TC-PTShi with the lowest PCL scores to match to the FS-PTShi group; and 42 TC-PTSlo men to better match demographics of FS-PTSlo.

Group comparisons for demographics and clinical characteristics are in Table 1. The four groups did not differ in gender, education, or partner relationship status. Both FS groups were older than TC-PTShi; FS-PTSlo was older than TC-PTSlo. Both FS groups included a lower proportion of non-white participants than TCs. FS-PTShi reported lower income than both TC groups; FS-PTSlo reported lower income than TC-PTSlo.

The two PTS-high groups (FS-PTShi, TC-PTShi) did not differ on the primary matching variable of PTS symptoms, nor in general mental health (MHI-5); both were higher than the two PTS-lo groups, who also did not differ. The same pattern was demonstrated for number of traumatic events (listed in Supplementary Tables 1, 2). FS-PTShi reported more dissociative symptoms than the other three groups, and TC-PTShi reported more dissociative symptoms than FS-PTSlo or TC-PTSlo (the latter two did not differ). Within FS, FS-PTShi reported younger FS onset age, and a non-significant trend toward greater seizure impact than FS-PTSlo. FS-PTSlo reported greater seizure severity than FS-PTShi. There were no group differences in FS condition duration, recency, or symptom frequency.

### 3.2. Descriptive results: correlations

Spearman correlations among variables are presented in Supplementary Tables 3, 4. Across participants, for most measures better socioemotional regulation was related to fewer clinical symptoms. There were few associations between socioemotional regulation and FS symptoms, with most for FS-PTShi.

### 3.3. Comparisons of groups’ socioemotional regulation

For the three hypotheses, those with FS and/or greater symptom load, namely FS-PTShi versus TC-PTShi, FS-PTSlo versus TC-PTSlo, and FS-PTShi versus FS-PTSlo, were expected to report greater difficulties on all socioemotional regulation measures. (Findings for affectionate touch frequency during waking and surrounding sleep were similar when examining only participants with a current partner.)

**Figure 1:**
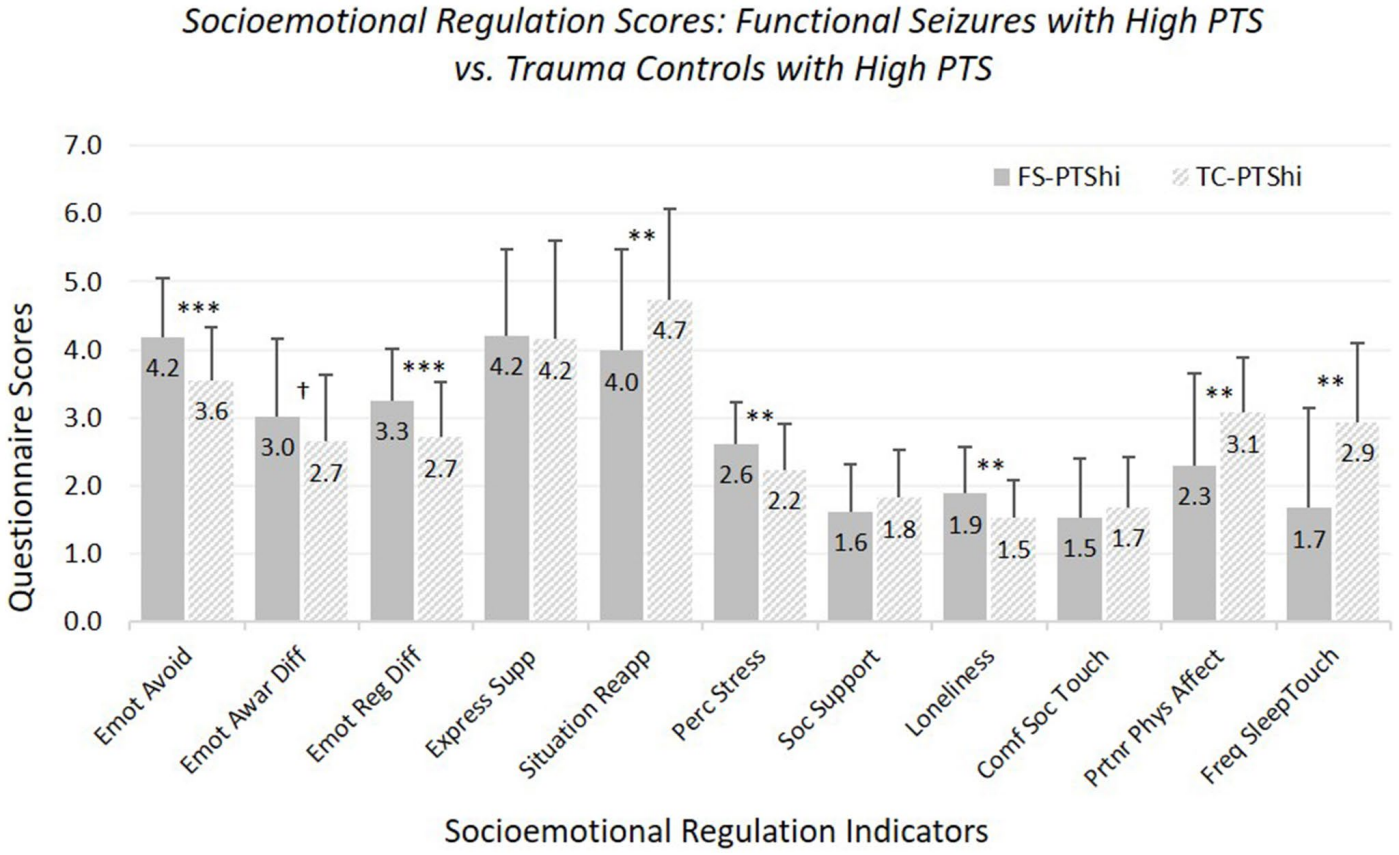
Means and standard deviations of socioemotional regulation characteristics: participants experiencing functional seizures and high posttraumatic stress symptoms versus trauma controls with high posttraumatic stress symptoms. Emot avoid = emotional avoidance per the Brief Experiential Avoidance Questionnaire (1–6 scale); emot awar diff = emotional awareness difficulties per the Difficulties in Emotion Regulation Scale (DERS) – awareness subscale (1–5 scale); emot reg diff = overall emotion regulation difficulties per the DERS without the awareness items (1–5 scale); express supp/situation reapp = expressive emotion suppression and situational reappraisal, the two subscales of the Emotion Regulation Questionnaire (1–7 scale); perc stress = perceived stress per the Perceived Stress Scale (0–4 scale); soc. support = social support per the Interpersonal Support Evaluation List (0–3 scale); loneliness = loneliness measured with the UCLA Loneliness Scale (0–3 scale); comf soc. touch = comfort with social touch per the Social Touch Questionnaire (0–4 scale); partnr phys affect = partner physical affection frequency per the Physical Affection Scale (0–4 scale); freq sleep touch = single item assessing frequency of touch with partner surrounding sleep (0–4 scale). ^†^*p* < 0.10. *p < 0.05. **p < 0.01. ***p < 0.001.

*Hypothesis 1:* FS-PTShi versus TC-PTShi (see Figure 1; Supplementary Table 5). For most measures, FS-PTShi reported greater difficulties than TC-PTShi: greater emotional avoidance, greater overall emotion regulation difficulties, less use of reappraisal, greater perceived stress, greater loneliness, and less frequent partner affectionate touch during waking and surrounding sleep (with a non-significant trend toward greater emotional awareness difficulties).

The two groups did not differ in expressive suppression, perceived social support, or social touch comfort. Age was a significant covariate for perceived social support, affectionate touch frequency, and sleep-touch; older age was associated with less of each.

Findings for FS-PTShi versus TC-PTShi were the same with our strict-inclusion sample, with exceptions that the trend-level difference for difficulties in emotional awareness reached significance, and the significant difference in physical affection with partner during waking became trend-level.

**Figure 2:**
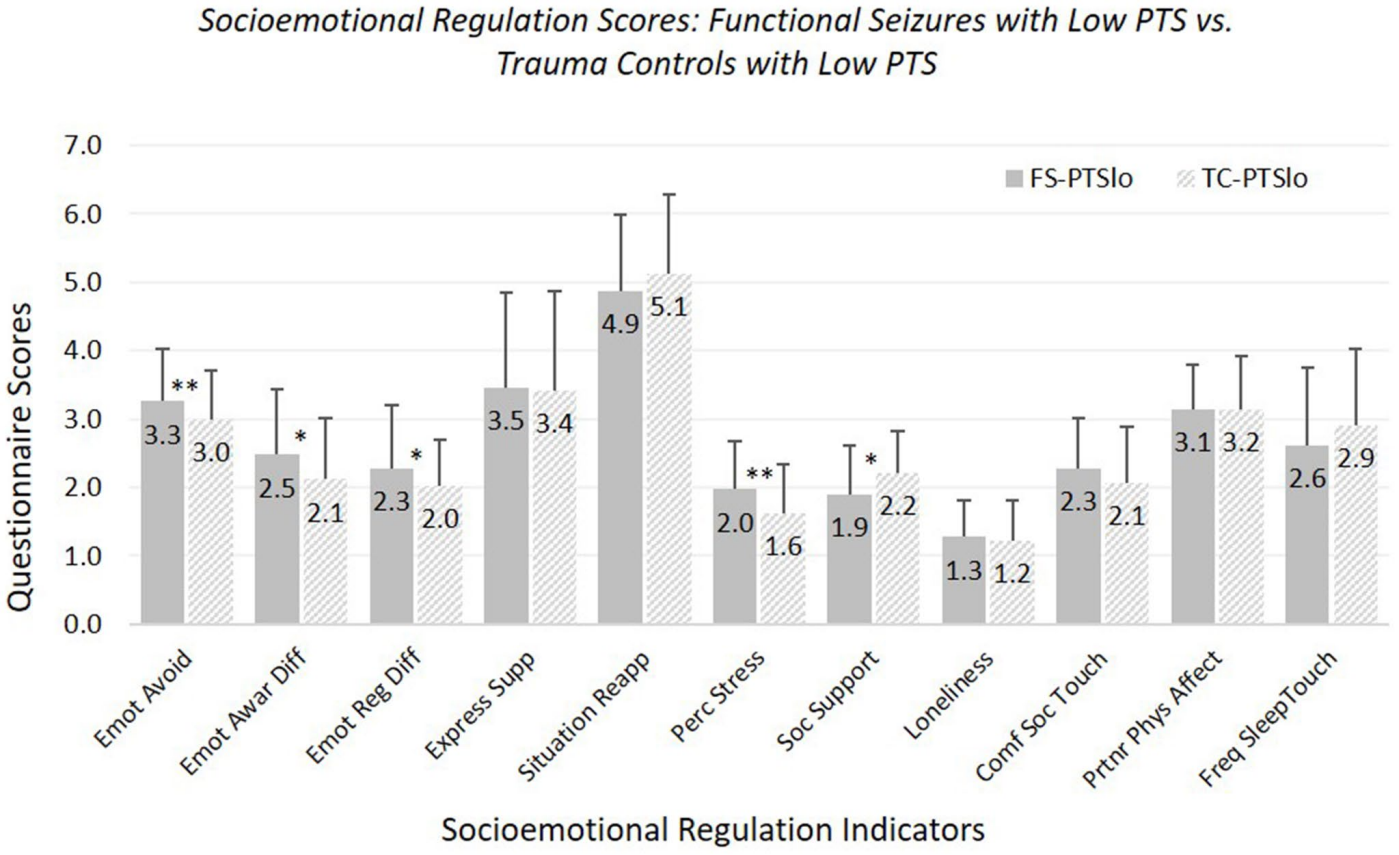
Means and standard deviations of socioemotional regulation characteristics: participants experiencing functional seizures and low posttraumatic stress symptoms versus trauma controls with low posttraumatic stress symptoms. Emot avoid = emotional avoidance per the Brief Experiential Avoidance Questionnaire (1–6 scale); emot awar diff = emotional awareness difficulties per the Difficulties in Emotion Regulation Scale (DERS) – awareness subscale (1–5 scale); emot reg diff = overall emotion regulation difficulties per the DERS without the awareness items (1–5 scale); express supp/situation reapp = expressive emotion suppression and situational reappraisal, the two subscales of the Emotion Regulation Questionnaire (1–7 scale); perc stress = perceived stress per the Perceived Stress Scale (0–4 scale); soc. support = social support per the Interpersonal Support Evaluation List (0–3 scale); loneliness = loneliness measured with the UCLA Loneliness Scale (0–3 scale); comf soc. touch = comfort with social touch per the Social Touch Questionnaire (0–4 scale); partnr phys affect = partner physical affection frequency per the Physical Affection Scale (0–4 scale); freq sleep touch = single item assessing frequency of touch with partner surrounding sleep (0–4 scale). ^†^*p* < 0.10. *p < 0.05. **p < 0.01.

*Hypothesis 2:* FS-PTSlo versus TC-PTSlo (see Figure 2; Supplementary Table 6). FS-PTSlo reported greater difficulties than TC-PTSlo on several measures (but fewer than for FS-PTShi versus TC-PTShi comparisons). FS-PTSlo reported greater emotional avoidance, emotional awareness difficulties, overall emotion regulation difficulties, and perceived stress, and less social support than TC-PTSlo.

Groups did not differ in use of expressive suppression or reappraisal, loneliness, comfort with social touch, or frequency of partner affectionate touch during waking or surrounding sleep. Age was a significant covariate for emotional avoidance, emotion regulation difficulties, and perceived stress (all fewer/less with older age), comfort with social touch (more with older age), and partner affectionate touch frequency (less with older age).

With our strict-inclusion sample, FS-PTSlo did not differ significantly from TC-PTSlo in emotional awareness difficulties, and the significant group difference in perceived social support became trend-level; patterns of significance for other measures remained the same.

**Figure 3:**
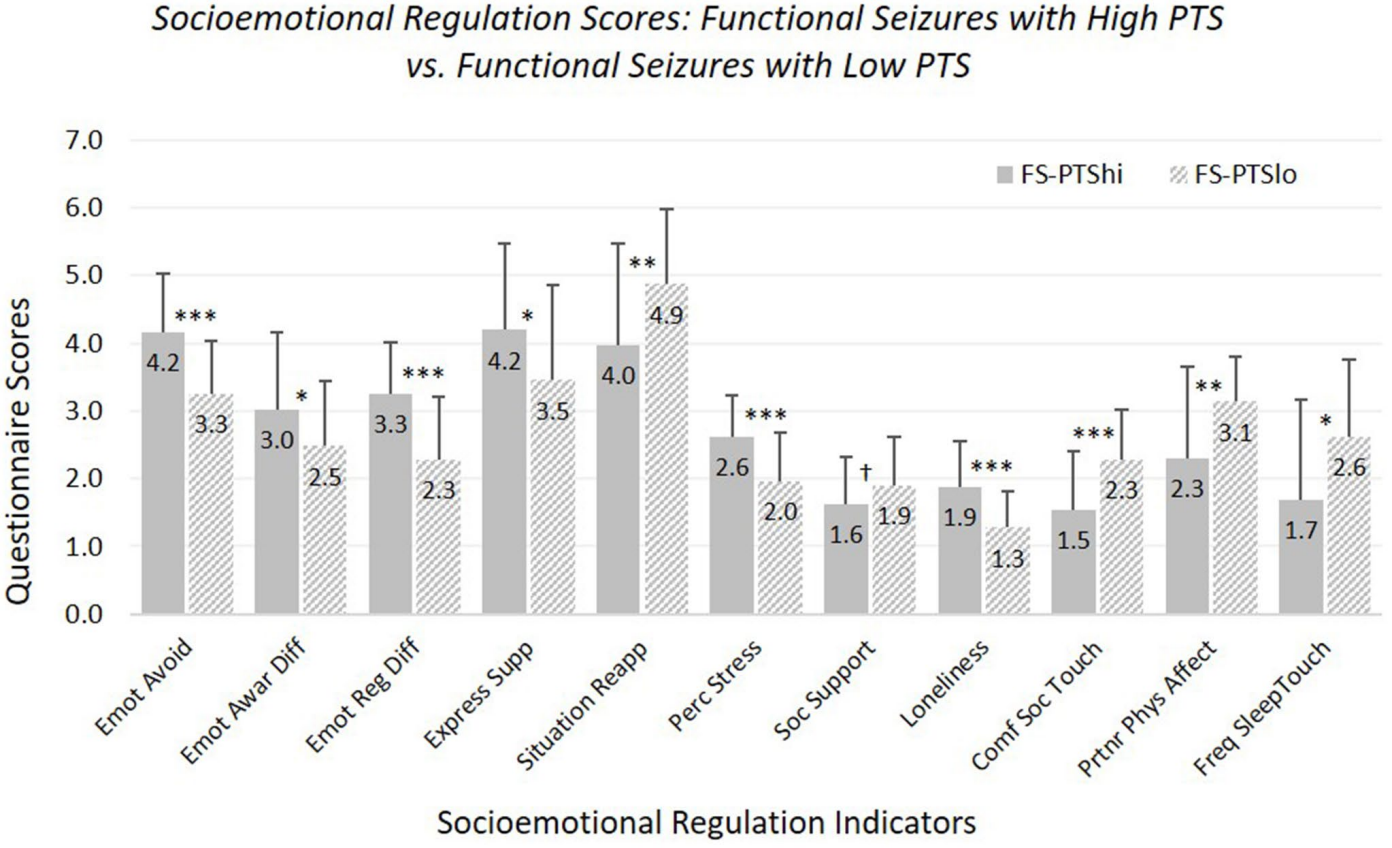
Means and standard deviations of emotional and social characteristics, contrasting participants with functional seizures experiencing high versus low posttraumatic stress symptoms. Emot avoid = emotional avoidance per the Brief Experiential Avoidance Questionnaire (1–6 scale); emot awar diff = emotional awareness difficulties per the Difficulties in Emotion Regulation Scale (DERS) – awareness subscale (1–5 scale); emot reg diff = overall emotion regulation difficulties per the DERS without the awareness items (1–5 scale); express supp/situation reapp = expressive emotion suppression and situational reappraisal, the two subscales of the Emotion Regulation Questionnaire (1–7 scale); perc stress = perceived stress per the Perceived Stress Scale (0–4 scale); soc. support = social support per the Interpersonal Support Evaluation List (0–3 scale); loneliness = loneliness measured with the UCLA Loneliness Scale (0–3 scale); comf soc. touch = comfort with social touch per the Social Touch Questionnaire (0–4 scale); partnr phys affect = partner physical affection frequency per the Physical Affection Scale (0–4 scale); freq sleep touch = single item assessing frequency of touch with partner surrounding sleep (0–4 scale). ^†^*p* < 0.10. *p < 0.05. **p < 0.01. ***p < 0.001.

*Hypothesis 3:* FS-PTShi versus FS-PTSlo (see Figure 3; Supplementary Table 7). FS-PTShi reported more difficulties than FS-PTSlo on 10 of 11 measures.

FS-PTShi and FS-PTSlo did not differ in perceived social support, although there was a non-significant trend toward less perceived support for FS-PTShi. Age was not a significant covariate for any measure except sleep-touch (older age associated with less sleep-touch).

Findings were the same with our strict-inclusion sample: differences were significant for all measures except social support.

## 4. Discussion

Functional seizures are complex neuropsychiatric conditions challenging to diagnose and treat. Their ongoing management is complicated by comorbidities that shape patients’ ability to navigate their internal and social world. We found based on an online survey that individuals with self-reported FS and prior trauma exposure reported greater socioemotional regulation difficulties than seizure-free trauma-exposed comparison groups with comparable levels of posttraumatic stress and mental health symptoms. This suggests problems experienced by those with FS were not accounted for by PTS level or poor mental health. Yet, while both FS groups reported more avoidance, emotion regulation difficulties, and stress than respective PTS-matched controls, only FS individuals with clinically-elevated PTS reported more loneliness and less affectionate touch in their partner relationships. Further, FS-PTShi reported more difficulties than FS-PTSlo on nearly all measures. Thus, clinically-elevated PTS may work synergistically with FS to exacerbate difficulties in intra-and interpersonal regulation domains.

### 4.1. Socioemotional self- and co-regulation in FS

#### 4.1.1. Emotion self-regulation

Emotional processes have long been thought to underpin FS and other functional disorders (77, 78) in FS/functional movement disorder]. Along with greater perceived stress and difficulties regulating distress, avoidance of inner emotional experience was most relevant in characterizing our FS sample. Emotional awareness findings were less consistent. Our emotional awareness measure assessed attention to one’s own feelings/emotions, which is only weakly related to alexithymia [difficulty identifying and describing feelings (79)], with which FS often is associated (80). FS participants did not suppress outward emotional displays more than TCs, unlike prior research versus healthy controls (15). This prior research used the Emotional Processing Scale, where items potentially combine experiential and expressive suppression [e.g., “bottled up emotions” (81)], whereas our measure [ERQ suppression subscale (69)] assessed only outward suppression. Thus, rather than outward display, profound experiential or emotional avoidance [e.g., of shame (41, 82, 83)]—whether linked to PTSD or intrinsic to FS—may serve as a vulnerability to, and/or proximal trigger for, dissociative and motor responses in FS (84). Further, FS-PTShi reported more dissociative symptoms than TC-PTShi, yet not more PTS nor overall mental health symptoms, echoing dissociation as implicated in FS phenomenology (85) and pathophysiology [(86); in other FND: (87)], particularly with comorbid FS and clinical PTS.

#### 4.1.2. Social (co-)regulation and affectionate touch

FS-PTShi reported more loneliness and less partner affectionate touch when awake and surrounding sleep than TC-PTShi, despite similar proportions in partner relationships. FS-PTShi and TC-PTShi did not differ, however, in perceived social support or comfort with social touch (nor ability to suppress emotion outwardly, noted above). We speculate social relationships and relationship attitudes more generally are not more compromised in FS, even when coupled with clinical PTS, than among those with clinical PTS alone, similar to prior work finding strong social network engagement in FS (22). Rather, more intimate experiences of physical and emotional connection may be affected in FS individuals with clinical PTS. Improving relationships and increasing affectionate touch during waking and/or surrounding sleep may yield treatment benefits, as physical touch may influence affect regulation and intersect with interoceptive processes (55), which are compromised in FS (88). Sleep-touch may be particularly beneficial, because it often occurs during low- or decreasing-arousal states, with fewer concurrent salient stimuli to distract from touch’s calming effects (53).

FS-PTSlo participants did not report less frequent affectionate touch in their partner relationships than controls. Rather, this important form of co-regulation was spared in this subgroup. FS-PTSlo also did not differ from controls in loneliness, perhaps suggesting a stable sense of social connection despite FS symptoms. The lack of association between socioemotional regulation and FS symptom frequency or severity (which, again, we interpret with caution) also suggests different mechanisms in FS-PTSlo.

### 4.2. Limitations and implications

Importantly, we relied on participants’ self-reported FS diagnostic history. Although we included participants in our FS groups based on careful examination of their answers to detailed quantitative and qualitative questions, we did not have verified diagnoses based on documentation of evaluations, providers’ direct reports, or clinical interviews. Findings were largely consistent when we re-tested our hypotheses with more strictly-defined diagnostic criteria based on EEG monitoring results, albeit self-reported. Limiting generalizability, our participants were mostly accepting of their diagnoses and able and willing to fill out a lengthy online survey. Participants recruited via FND-related social media sites may have had or desired high social support and connection.

To hold trauma exposure constant, we only included participants reporting prior trauma. Presence or absence of PTSD, and not trauma itself, has differentiated performance on cognitive and clinical measures (48); however, trauma exposure may have been a factor in ways not tested here. We also conceptualized FS as the primary diagnosis and PTSD as a secondary comorbidity; it is possible to conceptualize PTSD as primary in some cases, with FS as an additional feature. Given our small sample, particularly for FS-PTSlo, and unequal sample sizes between FS-PTSlo and TC-PTSlo, findings must be interpreted with caution. We did not consider patient subgroups besides high and low PTS. Given heterogeneity among FS, other subgroups may be fruitful to explore [e.g., high and low emotion dysregulation; (30, 31)].

As with any cross-sectional, self-report online survey, measures reflect participant experience (not behavior), and are colored by participant perceptions. Causality regarding socioemotional patterns and FS or PTS symptoms also cannot be inferred. Additionally, although we collected multiple individual measures that we grouped conceptually as indicators of “socioemotional regulation,” we chose to take a more granular analytical approach by conducting individual pairwise contrasts for each measure (rather than a multivariate analysis), for easier comparison with prior literature [e.g., (15, 22)].

In prior work we included FS participants with neurologist/epileptologist-verified diagnoses based on careful assessment including video EEG (41, 42); here, we chose to broaden our sample to FS individuals who may not have had access to epilepsy monitoring unit evaluation. Reasonable consistency between present findings and prior work, yet with some differences [e.g., fewer significant correlations between socioemotional processes and symptoms (49)—which also may be due to measure limitations (single items assessing seizure frequency and severity) and small sample size], suggests that a community sample may provide important information, alongside systematic comparisons with diagnostically-verified samples. Such an approach could facilitate understanding FS as a continuum, perhaps with subclinical presentations and normed self-report measures to capture them, akin to studies of depression and other clinical syndromes [e.g., (89, 90)].

### 4.3. What can we learn about FS without PTS?

As the field advances its knowledge of FND and PTSD intersections, it becomes both more challenging and more important to understand FS absent PTSD. This challenge is compounded by small samples, and uncertainty about past (traumatic) events when relying on self-report. Our findings nonetheless may have treatment implications for FS with non-clinical PTS. For example, FS-PTSlo were comparable to controls in using cognitive reappraisal as an emotion regulation strategy; this ability to think about a situation differently may help offset otherwise-detrimental effects of emotional avoidance and dysregulation and can be considered a potential relative strength. Concrete, problem-solving versus process-oriented approaches may be warranted, for example, to focus on lowering stress and increasing opportunities for social support. While close connection to others was not reported as problematic, whether those areas are spared, or reflect different views of interpersonal closeness, could be explored further.

## 5. Conclusion

In the present study, we identified social and emotion regulation challenges and strengths in FS individuals compared with PTS-matched controls. Those with FS indicated feeling comfortable with social touch at levels comparable to controls matched in PTS/mental health, but deriving a sense of physical and emotional closeness from intimate relationships appeared more challenging when posttraumatic stress symptoms were clinically elevated. Thus, PTSD-related disruptions in co-regulation may intensify self-regulation difficulties and avoidance tendencies in FS. Attention to social/interpersonal strategies for co-regulation alongside self-focused affect regulation may be an important avenue for understanding affective processes in FS and enhancing patients’ well-being.

## Data availability statement

The original contributions presented in the study are included in the article/Supplementary material, further inquiries can be directed to the corresponding author.

## Ethics statement

The studies involving human participants were reviewed and approved by Arizona State University’s Institutional Review Board. The participants provided their written informed consent to participate in this study.

## Author contributions

All authors listed have made a substantial, direct, and intellectual contribution to the work and approved it for publication.

## Conflict of interest

The authors declare that the research was conducted in the absence of any commercial or financial relationships that could be construed as a potential conflict of interest.

## Publisher’s note

All claims expressed in this article are solely those of the authors and do not necessarily represent those of their affiliated organizations, or those of the publisher, the editors and the reviewers. Any product that may be evaluated in this article, or claim that may be made by its manufacturer, is not guaranteed or endorsed by the publisher.
