# Supplementary material for: Socioemotional self- and co-regulation in functional seizures: comparing high and low posttraumatic stress
Source: Front Psychiatry. 2023 May 15;14:1135590. doi: 10.3389/fpsyt.2023.1135590 (PMC10225681; doi:10.3389/fpsyt.2023.1135590)
Supplement: Supplementary file 1 [file Table_1.pdf]

Supplementary Material

Roberts, N.A., Villarreal, L.D., & Burleson, M.H. (2023). Socioemotional Self- and Co-Regulation in Functional Seizures: Comparing High and Low Posttraumatic Stress. *Frontiers in Psychiatry.*

Contents:

Supplementary Participant Inclusion Information: Questions for determining self-reported diagnosis of functional seizures

Supplementary Table 1. *Trauma Events Reported, by Trauma Type: Number and Percentage by Participant Group*

Supplementary Table 2. *Trauma Event Reported as Most Significant, by Trauma Type: Number and Percentage by Participant Group*

Supplementary Table 3. *Spearman Correlations Between Clinical and Socioemotional Indicators Across the Full Sample*

Supplementary Table 4. *Spearman Correlations Between Socioemotional and Symptom Indicators for Functional Seizure Groups with High versus Low Levels of Posttraumatic Stress Symptoms*

Supplementary Table 5. *Means and Standard Deviations of Socioemotional Regulation Characteristics: Participants Experiencing Functional Seizures and High Posttraumatic Stress Symptoms Versus Trauma Controls with High Posttraumatic Stress Symptoms*

Supplementary Table 6. *Means and Standard Deviations of Socioemotional Regulation Characteristics: Participants Experiencing Functional Seizures and Low Posttraumatic Stress Symptoms Versus Trauma Controls with Low Posttraumatic Stress Symptoms*

Supplementary Table 7. *Means and Standard Deviations of Emotional and Social Characteristics, Contrasting Participants with Functional Seizures Experiencing High Versus Low Posttraumatic Stress Symptoms*

Supplementary Table 8. *Spearman Correlations Between Clinical and Socioemotional Indicators Across the Full Sample, with Stricter FS Diagnostic Inclusion for FS Participants*

Supplementary Table 9. *Spearman Correlations Between Socioemotional and Symptom Indicators for Functional Seizure Groups with High versus Low Levels of Posttraumatic Stress Symptoms: Stricter FS Diagnostic Inclusion Criteria Subsample*

Supplementary Table 10. *Means and Standard Deviations of Socioemotional Regulation Characteristics: Participants Experiencing Functional Seizures and High Posttraumatic Stress Symptoms Versus Trauma Controls with High Posttraumatic Stress Symptoms: Stricter FS Diagnostic Inclusion Criteria Subsample*

Supplementary Table 11. *Means and Standard Deviations of Socioemotional Regulation Characteristics: Participants Experiencing Functional Seizures and Low Posttraumatic Stress Symptoms Versus Trauma Controls with Low Posttraumatic Stress Symptoms: Stricter FS Diagnostic Inclusion Criteria Subsample*

Determining Self-reported Diagnosis of Functional Seizures

Summary: We included in our functional seizures (FS) group those who endorsed having seizures/seizure-like events and reported a diagnosis of FS, regardless of whether or not they agreed with the diagnosis. We augmented these criteria, with careful attention to survey respondents who did not complete the diagnosis item or who indicated a suspected or inconclusive FS diagnosis. We considered self-reported EEG results in those who had undergone EEG evaluation; we excluded anyone who had a clear diagnosis of epilepsy or other neurological condition without FS, and those for whom the EEG-based outcome was too unclear to ensure an FS diagnosis. We considered what name they preferred to call their seizures; what they reported their doctor said causes their seizures; medication; and open-ended fields where participants had the opportunity to write additional comments.

A diagnosis of FS was determined for the present study based on a combination of the following questions, which were included in the survey:

1. **Seizures are involuntary episodes of movements, sensations, or behaviors. Have you ever experienced a seizure or seizure-like episode before?** Yes No

2. **Please indicate your diagnostic history for each of the following:**

<u>Epilepsy</u>:

I have been diagnosed with this disorder and I AGREE with the diagnosis

I have been diagnosed with this disorder and I DISAGREE with the diagnosis

I suspect I have this disorder but have not been diagnosed

I do not have this disorder

Diagnoses/tests have been inconclusive

Other (please specify):

(Note: The above question in itself did not determine diagnostic group, as participants could experience mixed epileptic and functional seizures, but alongside responses to other questions, responses to this question helped clarify or indicated the diagnosis was not sufficiently clear to include in the present sample.)

<u>Functional Seizures</u>:

I have been diagnosed with this disorder and I AGREE with the diagnosis

I have been diagnosed with this disorder and I DISAGREE with the diagnosis

I suspect I have this disorder but have not been diagnosed

I do not have this disorder

Diagnoses/tests have been inconclusive

Other (please specify):

(Note: Participants who answered that they had been diagnosed with FS, or suspected they had FS, were included if responses to the other questions were supporting and not contradictory.)

3. **Do you believe that epilepsy...**

is part of your diagnosis

is NOT part of your diagnosis

Please explain: (open-ended textbox)

(Note: The above question in itself did not determine diagnostic group, as participants could experience mixed epileptic and functional seizures, but alongside responses to other questions, responses to this question helped clarify or indicated the diagnosis was not sufficiently clear to include in the present sample.)

4. **Have you ever had EEG monitoring of your seizures?** Yes No

If Yes:

Where did the EEG monitoring take place?

In an epilepsy monitoring unit

In another hospital setting

In a doctor's office

5. **What did the doctors determine, if anything, as a result of the EEG monitoring?**

Epilepsy

Non-epileptic seizures/attacks

Both epileptic and non-epileptic seizures/attacks (mixed)

Inconclusive

Other (please specify):

6. **What has YOUR DOCTOR said causes your seizures?** (open-ended textbox)

7. **What name do you prefer for your seizures?** (open-ended textbox)

8. **Do you have a sense of what FIRST led up to your seizures?** (open-ended textbox)

9. **Please list all current medications you are taking: (this helps us understand what doctors are prescribing for different conditions)**

(Note: This question did not determine diagnostic group, but if all other evidence suggested epilepsy, and the person was on antiepileptic medication, their responses were considered consistent with an epilepsy diagnosis and were not included in the FS group.)

Supplementary Table 1. *Trauma Events Reported, by Trauma Type: Number and Percentage by Participant Group*

	FS-PTShi		FS-PTSlo		TC-PTShi		TC-PTSlo	
Trauma event type	*N*	%	*N*	%	*N*	%	*N*	%
Natural disaster	6	11.54	0	0.00	16	17.58	16	12.80
Childhood physical / sexual abuse	38	73.08	22	57.89	56	61.54	43	34.40
Adult physical / sexual abuse	27	51.92	13	34.21	55	60.44	38	30.40
Danger of losing life / serious injury	26	50.00	9	23.68	31	34.07	28	22,40
Witnessing / learning about serious injury or death of someone	23	44.23	12	31.58	55	60.44	66	52.80
Serious accident	12	23.08	10	26.32	24	26.37	39	31.20
Violent crime	16	30.77	5	13.16	19	20.88	11	8.80
Sudden other event	37	71.15	15	39.47	50	54.95	57	45.60

Note. Events listed are from an adapted version of the Adverse Life Events Checklist. Participants could endorse multiple events.

Supplementary Table 2. *Trauma Event Reported as Most Significant, by Trauma Type: Number and Percentage by Participant Group*

Trauma event type	FS-PTShi		FS-PTSlo		TC-PTShi		TC-PTSlo	
	N	%	*N*	%	*N*	%	*N*	%
Experienced during childhood								
Sexual abuse	11	23.9	8	22.9	18	21.2	12	10.4
Physical abuse	5	10.9	5	14.3	6	7.1	5	4.3
Emotional abuse	2	4.3	3	8.6	1	1.2	0	0.0
Severe illness, injury, or threat	2	4.3	3	8.6	2	2.4	2	1.7
Grief/separation	4	8.7	4	11.4	2	2.4	14	12.2
Transportation accident	0	0.0	1	2.9	1	1.2	3	2.6
Military combat	0	0.0	0	0.0	0	0.0	0	0.0
Natural disaster	0	0.0	0	0.0	2	2.4	1	0.9
Witness to a traumatic event	0	0.0	0	0.0	1	1.2	4	3.5
Learning of a traumatic event	2	4.3	0	0.0	4	4.7	6	5.2
Other event	0	0.0	0	0.0	2	2.4	0	0.0
Experienced during adulthood								
Sexual abuse	5	10.9	1	2.9	12	14.1	12	10.4
Physical abuse	1	2.2	1	2.9	7	8.2	4	3.5
Emotional abuse	3	6.5	2	5.7	2	2.4	2	1.7
Severe illness, injury, or threat	3	6.5	3	8.6	3	3.5	7	6.1
Grief/separation	3	6.5	1	2.9	11	12.9	15	13.0
Transportation accident	0	0.0	0	0.0	0	0.0	7	6.1
Military combat	0	0.0	0	0.0	1	1.2	1	0.9
Natural disaster	0	0.0	0	0.0	0	0.0	3	2.6
Witness to a traumatic event	1	2.2	3	8.6	2	2.4	4	3.5
Learning of a traumatic event	3	6.5	0	0.0	6	7.1	7	6.1
Other event	1	2.2	0	0.0	2	2.4	6	5.2

Note. Events listed are from an adapted version of the Adverse Life Events Checklist and including additional events as reported by participants in an open-ended fashion, in response to a question about which event "stuck with them the most." Age of trauma event in years (*M*, *SD*) for FS-PTShi: 16.9 (12.6); for FS-PTSlo: 18.7 (14.9); for TC-PTShi: 18.6 (11.1); for TC-PTSlo: 20.2 (11.1).

Supplementary Table 3. *Spearman Correlations Between Clinical and Socioemotional Indicators Across the Full Sample*

Socioemotional indicator	PTS symptoms[a]	Mental health[b]	Dissociative symptoms[c]	Seizure frequency[d]	Seizure severity[d]	Seizure impact[e]
Emotional avoidance	.45***	.44***	.42***	.15	-.02	.13
Emotional awareness difficulties	.29***	.23***	.14*	.23*	-.02	.09
Emotion regulation difficulties	.52***	.62***	.46***	.05	-.01	.29*
Expressive suppression	.27***	.20***	.14*	.04	-.21	-.07
Situational reappraisal	-.24***	-.38***	-.17**	-.41***	-.17	-.14
Perceived stress	.52***	.70***	.42***	.16	.08	.37**
Social support	-.27***	-.36***	-.19**	-.14	.01	-.16
Loneliness	.38***	.47***	.28***	.18	-.09	.11
Comfort with social touch	-.33***	-.31***	-.26***	-.29**	-.01	-.03
Physical affection with partner	-.07	-.20**	-.03	-.01	.17	-.21
Frequency of sleep-touch	-.04	-.19**	.06	.23	.08	-.21

Note. PTS symptoms = PTSD Checklist for DSM-5; mental health = Mental Health Inventory-5; dissociative symptoms = Dissociative Experiences Scale II; seizure impact = Impact of Epilepsy Scale; seizure frequency, and severity = single items. Seizure impact, frequency, and severity were assessed only among FS participants.

[a]*n* = 218 to 297; [b]*n* = 211 to 290; [c]*n* = 210 to 289; [d]*n* = 57 to 82; [e]*n* = 47 to 68.

**p* < .05. ***p* < .01. ****p* < .001.

Supplementary Table 4. *Spearman Correlations Between Socioemotional and Symptom Indicators for Functional Seizure Groups with High versus Low Levels of Posttraumatic Stress Symptoms*

	Participant group					
	FS-PTShi			FS-PTSlo		
Socioemotional indicator	Seizure frequency[a]	Seizure severity[a]	Seizure impact[b]	Seizure frequency[c]	Seizure severity[c]	Seizure impact[d]
Emotional avoidance	.17	.07	.19	.05	.01	-.18
Emotional awareness difficulties	.34*	-.05	-.15	.08	.14	-.22
Emotion regulation difficulties	.18	.12	.32	-.19	-.01	.03
Expressive suppression	.06	-.27	-.18	-.03	-.03	-.09
Situational reappraisal	-.58***	-.40**	-.10	-.12	.07	-.11
Perceived stress	.14	.09	.32	.18	.31	.35*
Social support	-.16	-.11	-.17	-.09	.08	-.08
Loneliness	.21	.05	.11	.11	-.09	-.06
Comfort with social touch	-.31*	-.22	.03	-.22	.12	.15
Physical affection with partner	-.15	.05	-.15	.28	.15	-.06
Frequency of sleep-touch	.28	.00	-.07	.37	.01	-.08

Note. Seizure impact = Impact of Epilepsy Scale; seizure frequency and severity = single items.

[a]n = 30 to 46; [b]n = 23 to 35; [c]n = 26 to 36; [d]n = 23 to 33.

*$p < .05$. **$p < .01$. ***$p < .001$.

Supplementary Table 5. *Means and Standard Deviations of Socioemotional Regulation Characteristics: Participants Experiencing Functional Seizures and High Posttraumatic Stress Symptoms Versus Trauma Controls with High Posttraumatic Stress Symptoms*

	Participant group								
	FS-PTShi			TC-PTShi					
Socioemotional indicator	*n*	*M*	*SD*	*n*	*M*	*SD*	*F*(df)	*p*	η_p^2
Emotional avoidance	41	4.16	0.87	86	3.55	0.78	17.79 (1,124)	**<.001**	.125
Emotional awareness difficulties	42	3.02	1.14	86	2.65	0.97	3.18 (1,125)	.077	.025
Emotion regulation difficulties	43	3.25	0.77	86	2.71	0.82	13.38 (1,126)	**<.001**	.096
Expressive suppression	44	4.20	1.27	87	4.15	1.46	0.01 (1,128)	.911	.000
Situational reappraisal	44	3.98	1.50	87	4.74	1.33	9.24 (1,128)	**.003**	.067
Perceived stress	40	2.62	0.61	86	2.23	0.67	9.28 (1,123)	**.003**	.070
Social support	41	1.62	0.70	86	1.82	0.70	1.00 (1,124)	.320	.008
Loneliness	41	1.88	0.68	86	1.52	0.57	8.49 (1,124)	**.004**	.064
Comfort with social touch	44	1.54	0.86	86	1.68	0.74	1.01 (1,127)	.317	.008
Physical affection with partner	35	2.30	1.35	79	3.07	0.81	8.26 (1,111)	**.005**	.069
Frequency of sleep-touch	29	1.69	1.49	63	2.94	1.15	10.34 (1,89)	**.002**	.115

Note. **Bold font** signifies $p < .05$. FS-PTShi = functional seizures present, high post-traumatic stress scores; TC-PTShi = trauma controls (functional seizures not present), high post-traumatic stress scores; η_p^2 = partial eta-squared. Age was included as a covariate, and means are age-corrected.

Supplementary Table 6. *Means and Standard Deviations of Socioemotional Regulation Characteristics: Participants Experiencing Functional Seizures and Low Posttraumatic Stress Symptoms Versus Trauma Controls with Low Posttraumatic Stress Symptoms*

	Participant group								
	FS-PTSlo			TC-PTSlo					
Socioemotional indicator	*n*	*M*	*SD*	*n*	*M*	*SD*	*F*(df)	*p*	η_p^2
Emotional avoidance	34	3.26	0.77	123	2.99	0.72	7.61 (1,154)	**.006**	.047
Emotional awareness difficulties	33	2.48	0.96	124	2.12	0.90	3.95 (1,154)	**.049**	.025
Emotion regulation difficulties	33	2.28	0.93	124	2.03	0.66	6.55 (1,154)	**.011**	.041
Expressive suppression	34	3.46	1.39	125	3.42	1.46	0.09 (1,156)	.767	.001
Situational reappraisal	34	4.87	1.12	125	5.13	1.15	2.10 (1,156)	.150	.013
Perceived stress	34	1.97	0.71	123	1.63	0.71	10.03 (1,154)	**.002**	.061
Social support	34	1.90	0.72	122	2.21	0.62	6.80 (1,153)	**.010**	.043
Loneliness	34	1.28	0.53	122	1.23	0.57	0.84 (1,153)	.360	.005
Comfort with social touch	34	2.28	0.73	125	2.07	0.81	0.15 (1,156)	.696	.001
Physical affection with partner	29	3.14	0.66	113	3.15	0.77	0.11 (1,139)	.742	.001
Frequency of sleep-touch	26	2.62	1.13	97	2.91	1.11	0.86 (1,120)	.356	.007

Note. **Bold font** signifies $p < .05$. FS-PTSlo = functional seizures present, low post-traumatic stress scores; TC-PTSlo = functional seizures not present, low post-traumatic stress scores; η_p^2 = partial eta-squared. Age was included as a covariate, and means are age-corrected.

Supplementary Table 7. *Means and Standard Deviations of Emotional and Social Characteristics, Contrasting Participants with Functional Seizures Experiencing High Versus Low Posttraumatic Stress Symptoms*

	Participant group								
	FS-PTShi			FS-PTSlo					
Socioemotional indicator	*n*	*M*	*SD*	*n*	*M*	*SD*	*F*(df)	*p*	η_p^2
Emotional avoidance	41	4.16	0.87	34	3.26	0.77	19.38 (1,72)	**<.001**	.212
Emotional awareness difficulties	42	3.02	1.14	33	2.48	0.96	5.13 (1,72)	**.027**	.066
Emotion regulation difficulties	43	3.25	0.77	33	2.28	0.93	22.93 (1,73)	**<.001**	.239
Expressive suppression	44	4.20	1.27	34	3.46	1.39	5.83 (1,75)	**.018**	.072
Situational reappraisal	44	3.98	1.50	34	4.87	1.12	7.97 (1,75)	**.006**	.096
Perceived stress	40	2.62	0.61	34	1.97	0.71	16.99 (1,71)	**<.001**	.193
Social support	41	1.62	0.70	34	1.90	0.72	3.50 (1,72)	.065	.046
Loneliness	41	1.88	0.68	34	1.28	0.53	16.04 (1,72)	**<.001**	.182
Comfort with social touch	44	1.54	0.86	34	2.28	0.73	14.41 (1,75)	**<.001**	.161
Physical affection with partner	35	2.30	1.35	29	3.14	0.66	9.88 (1,61)	**.003**	.139
Frequency of sleep-touch	29	1.69	1.47	26	2.62	1.13	7.16 (1,52)	**.010**	.121

Note. **Bold font** signifies $p < .05$. FS-PTShi = functional seizures present, high post-traumatic stress scores; FS-PTSlo = functional seizures present, low post-traumatic stress scores; η_p^2 = partial eta-squared. Age was included as a covariate, and means are age-corrected.

Supplementary Table 8. *Spearman Correlations Between Clinical and Socioemotional Indicators Across the Full Sample, with Stricter FS Diagnostic Inclusion for FS Participants*

Socioemotional indicator	PTS symptoms[a]	Mental health[b]	Dissociative symptoms[c]	Seizure frequency[d]	Seizure severity[d]	Seizure impact[e]
Emotional avoidance	.47***	.44***	.43***	.21	-.09	.17
Emotional awareness difficulties	.30***	.23***	.15*	.20	-.25	-.10
Emotion regulation difficulties	.52***	.50***	.46***	.16	-.07	.30*
Expressive suppression	.30***	.20***	.11	.08	-.38**	-.14
Situational reappraisal	-.25***	-.34***	-.20***	-.46***	-.16	-.24
Perceived stress	.52***	.61***	.41***	.33*	-.01	.44**
Social support	-.31***	-.38***	-.28***	-.11	-.02	-.01
Loneliness	.37***	.42***	.28***	.22	-.10	.22
Comfort with social touch	-.33***	-.30***	-.25***	-.45***	-.10	-.15
Physical affection with partner	-.05	-.17*	-.02	-.03	.00	-.38*
Frequency of sleep-touch	.00	-.13	.11	.29	-.04	-.19

Note. PTS symptoms = PTSD Checklist for DSM-5; mental health = Mental Health Inventory-5; dissociative symptoms = Dissociative Experiences Scale II; seizure impact = Impact of Epilepsy Scale; seizure frequency, and severity = single items. Seizure impact, frequency, and severity were assessed only among FS participants.

[a]n = 196 to 265; [b]n = 192 to 260; [c]n = 191 to 259; [d]n = 34 to 50; [e]n = 29 to 43.

*$p \leq .05$. **$p < .01$. ***$p < .001$.

Supplementary Table 9. *Spearman Correlations Between Socioemotional and Symptom Indicators for Functional Seizure Groups with High versus Low Levels of Posttraumatic Stress Symptoms: Stricter FS Diagnostic Inclusion Criteria Subsample*

	Participant group					
	FS-PTShi			FS-PTSlo		
Socioemotional indicator	Seizure frequency[a]	Seizure severity[a]	Seizure impact[b]	Seizure frequency[c]	Seizure severity[c]	Seizure impact[d]
Emotional avoidance	.07	-.03	-.01	.16	-.12	-.05
Emotional awareness difficulties	.18	-.35	-.32	.23	.04	-.06
Emotion regulation difficulties	.14	-.02	.29	.01	-.09	.07
Expressive suppression	-.10	-.46*	-.36	.20	-.14	-.13
Situational reappraisal	-.61***	-.40*	-.36	-.17	.11	.10
Perceived stress	.25	.04	.32	.41	.18	.43
Social support	-.01	-.13	-.07	-.19	.08	.11
Loneliness	.23	.08	.23	.07	-.30	.01
Comfort with social touch	-.46*	-.28	.06	-.33	.18	.02
Physical affection with partner	-.28	-.17	-.51*	.46*	.04	-.07
Frequency of sleep-touch	.54*	-.02	.14	.44	-.22	-.15

Note. Seizure impact = Impact of Epilepsy Scale; seizure frequency and severity = single items.

[a]n = 21 to 28; [b]n = 18 to 23; [c]n = 17 to 22; [d]n = 15 to 20.

*$p < .05$. **$p < .01$. ***$p < .001$.

Supplementary Table 10. *Means and Standard Deviations of Socioemotional Regulation Characteristics: Participants Experiencing Functional Seizures and High Posttraumatic Stress Symptoms Versus Trauma Controls with High Posttraumatic Stress Symptoms: Stricter FS Diagnostic Inclusion Criteria Subsample*

	Participant group								
	FS-PTShi			TC-PTShi					
Socioemotional indicator	*n*	*M*	*SD*	*n*	*M*	*SD*	*F*(df)	*p*	η_p^2
Emotional avoidance	25	4.41	0.83	86	3.55	0.78	26.24 (1,108)	**<.001**	.195
Emotional awareness difficulties	25	3.23	1.26	86	2.65	0.97	5.64 (1,108)	.019	.050
Emotion regulation difficulties	26	3.47	0.77	86	2.71	0.82	19.75 (1,109)	**<.001**	.153
Expressive suppression	26	4.26	1.25	87	4.15	1.46	0.16 (1,110)	.691	.001
Situational reappraisal	26	3.75	1.37	87	4.74	1.33	11.39 (1,110)	**.001**	.094
Perceived stress	25	2.75	0.64	86	2.23	0.67	11.56 (1,108)	**<.001**	.097
Social support	25	1.67	0.80	86	1.82	0.70	0.02 (1,108)	.892	.000
Loneliness	25	1.92	0.76	86	1.52	0.57	5.18 (1,108)	**.025**	.046
Comfort with social touch	26	1.49	0.99	86	1.68	0.74	0.97 (1,109)	.327	.009
Physical affection with partner	20	2.43	1.38	79	3.07	0.81	3.07 (1,96)	.083	.031
Frequency of sleep-touch	16	1.69	1.66	63	2.94	1.15	6.82 (1,76)	**.011**	.082

Note. **Bold font** signifies $p < .05$. FS-PTShi = functional seizures present, high post-traumatic stress scores; TC-PTShi = trauma controls (functional seizures not present), high post-traumatic stress scores; η_p^2 = partial eta-squared. Age was included as a covariate, and means are age-corrected.

Supplementary Table 11. *Means and Standard Deviations of Socioemotional Regulation Characteristics: Participants Experiencing Functional Seizures and Low Posttraumatic Stress Symptoms Versus Trauma Controls with Low Posttraumatic Stress Symptoms: Stricter FS Diagnostic Inclusion Criteria Subsample*

	Participant group								
	FS-PTSlo			TC-PTSlo					
Socioemotional indicator	*n*	*M*	*SD*	*n*	*M*	*SD*	*F*(df)	*p*	η_p^2
Emotional avoidance	21	3.23	0.78	123	2.99	0.72	4.46 (1,141)	**.036**	.031
Emotional awareness difficulties	20	2.47	0.90	124	2.12	0.90	2.64 (1,141)	.106	.018
Emotion regulation difficulties	20	2.33	0.97	124	2.03	0.66	5.91 (1,141)	**.016**	.040
Expressive suppression	21	3.21	1.41	125	3.42	1.46	0.23 (1,143)	.632	.002
Situational reappraisal	21	4.98	1.13	125	5.13	1.15	0.83 (1,143)	.364	.006
Perceived stress	20	1.99	0.51	123	1.63	0.71	7.91 (1,140)	**.006**	.053
Social support	21	1.97	0.70	122	2.21	0.62	3.20 (1,140)	.076	.022
Loneliness	21	1.25	0.58	122	1.23	0.57	0.34 (1,140)	.560	.002
Comfort with social touch	21	2.31	0.75	125	2.07	0.81	0.06 (1,143)	.803	.000
Physical affection with partner	18	3.27	0.56	113	3.15	0.77	1.28 (1,128)	.261	.010
Frequency of sleep-touch	17	2.65	1.17	97	2.91	1.11	0.32 (1,111)	.571	.003

Note. **Bold font** signifies $p < .05$. FS-PTSlo = functional seizures present, low post-traumatic stress scores; TC-PTSlo = functional seizures not present, low post-traumatic stress scores; η_p^2 = partial eta-squared. Age was included as a covariate, and means are age-corrected.

Supplementary Table 12. *Means and Standard Deviations of Emotional and Social Characteristics, Contrasting Participants with Functional Seizures Experiencing High Versus Low Posttraumatic Stress Symptoms: Stricter FS Diagnostic Inclusion Criteria Subsample*

	Participant group								
	FS-PTShi			FS-PTSlo					
Socioemotional indicator	*n*	*M*	*SD*	*n*	*M*	*SD*	*F*(df)	*p*	η_p^2
Emotional avoidance	25	4.41	0.83	21	3.23	0.78	22.22 (1,43)	**<.001**	.341
Emotional awareness difficulties	25	3.23	1.26	20	2.47	0.90	5.22 (1,42)	**.028**	.110
Emotion regulation difficulties	26	3.47	0.77	20	2.33	0.97	18.27 (1,43)	**<.001**	.298
Expressive suppression	26	4.26	1.25	21	3.21	1.41	7.12 (1,44)	**.011**	.139
Situational reappraisal	26	3.75	1.37	21	4.98	1.13	9.85 (1,44)	**.003**	.183
Perceived stress	25	2.75	0.64	20	1.99	0.51	18.11 (1,42)	**<.001**	.301
Social support	25	1.67	0.80	21	1.97	0.70	2.31 (1,43)	.136	.051
Loneliness	25	1.92	0.76	21	1.25	0.58	10.82 (1,43)	**.002**	.201
Comfort with social touch	26	1.49	0.99	21	2.31	0.75	8.72 (1,44)	**.005**	.165
Physical affection with partner	20	2.43	1.38	18	3.27	0.56	6.95 (1,35)	**.012**	.166
Frequency of sleep-touch	16	1.69	1.66	17	2.65	1.17	4.24 (1,30)	**.048**	.124

Note. **Bold font** signifies $p < .05$. FS-PTShi = functional seizures present, high post-traumatic stress scores; FS-PTSlo = functional seizures present, low post-traumatic stress scores; η_p^2 = partial eta-squared. Age was included as a covariate, and means are age-corrected.
